# Lessons Learned: Characterizing Emergency Department Disposition Conversations for Veterans with Dementia Using Direct Observations

**DOI:** 10.1002/alz70858_100538

**Published:** 2025-12-25

**Authors:** Justine Seidenfeld, Matthew Tucker, Melissa Harris‐Gersten, Gemmae M Fix, Nina R Sperber, Susan Nicki Hastings

**Affiliations:** ^1^ Durham VA Healthcare System, Durham, NC, USA; ^2^ VA Bedford Healthcare System, Bedford, MA, USA

## Abstract

The emergency department (ED) disposition decision carries significant implications for patient outcomes and care experiences. However, little is known about how people living with dementia and their care partners participate in these decisions. This GEAR 2.0 ADC‐funded ethnographic study employed direct observation methods, guided by a semi‐structured data collection tool, to better understand how ED disposition conversations unfold in real‐world settings. Over 45 days of screening, we recruited 20 patient and care partner dyads, observing all interactions and conversations with their ED providers during their ED visit.

We will present lessons learned regarding the feasibility and acceptability of recruiting people living with dementia and their care partners in the ED. These lessons include overcoming challenges in real‐time screening for eligible participants through electronic health records and balancing the timing of recruitment to integrate into ED workflow without overburdening staff. Patient and public involvement—particularly through a community advisor group associated with the GEAR 2.0 ADC program—was pivotal in shaping recruitment methods, such as determining the ideal timing to approach patients and care partners.

We will also examine ED‐specific study design considerations for assessing participant capacity to consent and assent, as well as obtaining proxy caregiver consent. Additionally, we will discuss insights about selecting survey instruments to measure dementia severity and caregiver burden, emphasizing the practical nuances of using these tools in a fast‐paced ED environment.

Finally, we will highlight our experience of using ethnographic methods to capture rich, holistic data on the experiences of people living with dementia and their care partners, and how this can be applied to other studies to offer a deeper understanding of ED care.

